# Design and assessment of a public health course as a general education elective for non-medical undergraduates

**DOI:** 10.3389/fpubh.2025.1496283

**Published:** 2025-02-17

**Authors:** Xinyang Li, Xiaoxi Zheng, Boqiang Wen, Bowei Zhang, Xiaolong Xing, Liye Zhu, Wentao Gu, Shuo Wang

**Affiliations:** ^1^Tianjin Key Laboratory of Food Science and Health, Research Institute of Public Health, School of Medicine, Nankai University, Tianjin, China; ^2^School of Public Health, Jilin University, Changchun, China; ^3^Institute of Health Education, Hebei Provincial Center for Disease Control and Prevention, Shijiazhuang, China

**Keywords:** public health, pedagogy, non-medical students, undergraduate, general education curriculum, instruction

## Abstract

Although public health programs among undergraduate students have been increasing and gaining popularity worldwide, few studies have focused on the needs and structure of public health courses for non-medical students. This study aimed to design a public health course as a general education elective for non-medical undergraduates at Nankai University, one of China’s leading multidisciplinary and research-oriented universities. Students’ feedback on the course was collected and analyzed after the completion of the elective course. We designed and developed the course under the general education elective course at Nankai University. The course includes four segments: (a) Public Health Fundamentals and Population Research Methods; (b) Chemical Safety and Health, (c) Diet, Nutrition, and Health, (d) Immunology, Microbiology, and Infectious Diseases, spanning 34 class hours (with 6 class hours designated for a flipped classroom format). The teaching content was divided into five parts: (1) Health and Medicine Knowledge, (2) Public Health Knowledge, (3) Public Health Methodology and Philosophy, (4) Proper View of Health Issues, and (5) Values Education. Students’ feedback after the course indicated that Diet, Nutrition, and Health was the most interesting segment, and the students considered basic biological or medical knowledge to be more important than other public health knowledge. A problem-based learning model was implemented for flipped classrooms, and we found that the problem-based learning questions were not only helpful for students’ knowledge construction but also for educators in understanding and managing the learning expectations of non-medical students. We believe that the lesson may guide other institutions in designing similar curricula.

## 1 Introduction

Public health is a multidisciplinary field that encompasses a wide range of scientific and practical approaches aimed at improving population health (1, 2). Public health education is typically targets at graduate students who have finished their undergraduate studies in various fields such as medicine, nursing, biology, sociology, and environmental science (3). However, a significant shift has been taking place in recent years, as more undergraduate programs are incorporating public health courses into their curricula (4, 5). This trend is partly driven by the acknowledgment of the diverse specializations and the wide range of job opportunities that exist within the public health field, which encompasses everything from epidemiology and health policy to environmental health and global health initiatives (6). Furthermore, in the wake of the COVID-19 pandemic, there has been an increasing emphasis on the importance of public health (7, 8). The global health crisis has prompted people from all walks of life to seek a deeper understanding of health issues related to pandemic prevention policies. As a result, there is a growing demand for students to gain knowledge in public health, not only to pursue careers in the field but also to make informed decisions that impact their own health and the wellbeing of their communities (9). The incorporation of public health courses into undergraduate programs reflects a wider educational response to the challenges posed by the pandemic (7). This reflects a trend that students should prepare early in their academic careers for the complexities of public health crises and the multifaceted nature of health outcomes. By teaching undergraduates the fundamentals of public health, universities enable students to contribute more effectively to society by equipping with the knowledge and skills necessary to engage with and address the contemporary health challenges (10). This trend represents a positive evolution in health education, one that is likely to produce a generation of more health-literate citizens for future public health (11, 12). So far, more and more universities have focused on public health curricula in undergraduate, even in some countries where public health used to be graduate education for medical-related majors, such as Brazil, Singapore, and the United States (10, 13, 14).

In China, it is worth noting that the approach may differ as public health has long been performed at the undergraduate level, but considered a vocational education. The traditional pathway to public health expertise has been through specialized education at both the undergraduate and graduate levels, where in-depth courses are usually available to students majoring in public health or preventive medicine (15). Most studies on public health education so far still focused on professional education or as an integral part of medical students’ education (16, 17). Although there might be public health courses for non-medical undergraduates, few studies have particularly focused on or investigated the public health courses for non-medical undergraduates. How non-medical undergraduate students perceive and value a public health course remains an issue that few pay particular attention.

The general course is a crucial component of the undergraduate education system, aiming to promote the holistic development of students by cultivating healthy values, integrated knowledge perspectives, comprehensive personal qualities, and innovative practical abilities (18). General education focuses on fostering students’ overall growth, with an emphasis on enhancing capability and innovation skills. Adhering to moral education and undergraduate-centered principles, it emphasizes reform, innovation, and collaborative education (19). The curriculum consists of required and elective general courses. Elective general courses extend beyond the required ones, facilitating interdisciplinary knowledge integration, promoting diverse cognitive frameworks, and cultivating a broad cognitive vision (20, 21). The general course prepares students to adapt to societal changes and become constructors and creators of future civilizations (22). Including a public health course in the general curriculum offers a valuable opportunity for non-medical undergraduates, enabling them to acquire essential public health insights into public health and apply interdisciplinary knowledge to real-world challenges.

This article elaborates on a public health curriculum specifically designed for non-medical undergraduate students as a general education elective course. The course was offered for a semester, from February 2024 to June 2024. On completion, the university conducted an end-of-term evaluation. Upon completion, the university conducted an end-of-term evaluation. Also, students’ feedback on the elective course was collected using a self-reported questionnaire for the assessment of the course.

## 2 Overview of the pedagogy

Audience: This course is designed for undergraduate students in the entire university and is a general elective course. The course is intended without prerequisite requirements, ensuring its content is accessible and relevant to undergraduate students from various academic levels and diverse educational backgrounds seeking knowledge in public health. This course provides a teaching outline, a schedule during the course selection stage, and an “Overview of Public Health” section in the first class. Through a systematic and comprehensive introduction to public health and this course, along with a public health perspective on the characteristics and health issues within the discipline, students can gain an understanding of the concepts, thought processes, and challenges of this course, and make timely adjustments in subsequent chapters of teaching.

Teacher–student interaction: Public health is an interdisciplinary application of medicine, biology, mathematics and statistics, chemistry, sociology, management, law, and other disciplines. This course is also a general elective, so it focuses on interaction with students, guiding them to think actively, linking theory with practice, and integrating multiple disciplines in the teaching curriculum. The flipped classroom is an innovative pedagogical approach in which students engage with instructional content outside of the classroom, as presented in class (23, 24). Here in the course, a flipped classroom was set during the class, with students preparing topics of interest related to public health in advance. Considering that students lack a background in biomedical sciences, the course’s flipped classrooms provided two options for participation for them. They could either (1) submit a Problem-Based Learning (PBL) question related to public health to the teacher, along with the rationale for the asking or a discussion about it, or (2) work in groups of 1–5 people to perform a presentation on issues of public health. By doing this, teachers can grasp the public health topics that students are truly interested in and understand their real needs, which is also more readily acceptable and less of a struggle for non-medical undergraduates than mandatory presentations for all students.

Teaching objectives: The overall teaching objectives of this course are divided into (1) knowledge objectives, (2) ability objectives, and (3) attitude and values education objectives.

### 2.1 Knowledge objective

The general knowledge objective of the course is to comprehend the essence, historical evolution, and cutting-edge advancements in public health while also examining health methodologies from a collective perspective. It encompasses specialized knowledge in areas like food hygiene, nutrition, infectious diseases and immunology, preventive medicine, and environmental health, among others. Students should become familiar with the research paradigms, operational characteristics, and methodologies associated with public health studies, keeping up with the latest scholarly developments in the field.

### 2.2 Ability objectives

The ability objectives focus on nurturing critical thinking and analytical skills in students from a public health perspective, empowering them to tackle public health issues and specific health issues with scientific rigor and objectivity. This includes equipping students with the knowledge needed to analyze and propose solutions to public health challenges. Additionally, the course aims to enhance communication, collaboration, and problem-solving skills through interactive discussions. Overall, the goals emphasize fostering a scientific mindset toward public health, promoting interdisciplinary integration concepts, and developing effective communication and collaborative skills.

### 2.3 Attitude and values education

The objective of attitude and values education is to establish a sense of responsibility for public health and individual wellbeing within group settings through the study of this course. It aims to cultivate scientific thinking and an innovative spirit, encouraging students to address public health issues with a scientific approach. Additionally, it seeks to develop a deep understanding of the importance and significance of the public health system, fostering awareness of “full cycle health.” The course also emphasizes strengthening social responsibility and establishing a moral code of conduct grounded in public health principles.

The course design has 17 sessions and 34 class hours, which is divided into four teaching segments:

Public Health Fundamentals and Population Research Methods;
Chemical Safety and Health;
Diet, Nutrition, and Health;
Immunology, Microbiology, and Infectious Diseases.

According to the classification of teaching content, the teaching content is divided into five modules: (1) Health and Medicine Knowledge; (2) Public Health Knowledge; (3) Public Health Methodology and Philosophy; (4) Proper View on Health Issues; (5) Values Education. The module on “Health and Medicine Knowledge” is the knowledge on basic health and medicine, which focuses on personal health and is fundamental to public health; the module on “Public Health Knowledge” includes knowledge on public health and preventive medicine, such as the knowledge on healthy lifestyle and diet, environmental health, immune, and vaccine; the module on “Public Health Methodology and Philosophy” includes the statistic and population-based research, health economic evaluation, and way of thinking from the perspective of the entire population, etc.; the module on “Proper View on Health Issues” focused on hotpots of current issues, such as “how to properly understand the possible link between height and cancer,” and “how to accurately approach the risks of food additives”; the module on “Values Education” includes “prevention first,” “balanced diet,” and “food conservation,” etc. The detailed teaching arrangement and teaching content are shown in Table 1.

**Table 1:** The course content and teaching arrangement of the public health elective general course.

Segments	Course name	Class hours	Teaching methods	Teaching content*
Public Health Fundamentals and Population Research Methods	Overview of Public Health	2	Teaching	2, 3, and 5
	History and Frontiers of Public Health	2	Teaching	1, 2, 3, and 5
	Introduction to Preventive Medicine	2	Teaching	2 and 3
	Research Methods for Population-based study	2	Teaching	1–4
	Healthy Lifestyle and Diseases	2	Teaching	1–4
	Group Presentation and Discussion (1)	2	Flipped classroom	
Chemical Safety and Health	Chemical Pollution and Prevention	2	Teaching	1, 2, and 4
	Environmental Hygiene and Health	2	Teaching	1, 2, 4, and 5
	The Concept and Content of Food Safety	2	Teaching	2, 4, and 5
Diet, nutrition, and Health	Nutrients and the Nutritional Value of Food	2	Teaching	1–5
	Nutrition and Disease	2	Teaching	1, 4, and 5
	Dietary Guidelines for Chinese Residents	2	Teaching	1–4
	Group Presentation and Discussion (2)	2	Flipped classroom	
Immunology, Microbiology, and Infectious Diseases.	Microorganisms and Immunity	2	Teaching	1,4
	Antibodies and Vaccines	2	Teaching	1,3,4,5
	Bacterial Food Poisoning	2	Teaching	1,2,3
	Group Presentation and Discussion (3)	2	Flipped classroom	

*Teaching content: 1. Health and Medicine Knowledge; 2. Public Health Knowledge; 3. Public Health Methodology and Philosophy; 4. Proper View on Health Issues; and 5. Values Education.

## 3 Settings and students

Nankai University is a comprehensive university with a wide range of disciplines covering the arts, sciences, engineering, and medicine. Currently (as of January 2024), there are 16,372 undergraduate students enrolled in the university. The Medical School of Nankai University was officially established in 1988 and currently offers four undergraduate programs: Clinical Medicine, Stomatology, Intelligent Medical Engineering, and Ophthalmology and Optometry, with no majors or degrees in public health available at the undergraduate, master’s, or doctoral levels. Apart from the 837 students in the medical school, all other undergraduate students are eligible to take this course as an elective course.

The course size for an elective general course at Nankai University is usually small to medium, with a limit of no more than 50 and no fewer than 5 undergraduates. Students can freely add or drop the course before the start of the class, or in the first 3 weeks after the course begins, and are allowed to withdraw from the course at the mid-term week (the ninth week).

The grading system for this general elective course is binary: pass/fail. The grading criteria consist of ① attendance, ② group presentations or question submission, and ③ a paper of discussion, and related to public health or suggestion on the class as a final assignment.

## 4 Results and assessment

The course started on 21 February 2024. Fifty students chose the elective at the onset, and there was one student dropout at the mid-term week. The remaining 49 students from 22 different non-medical majors, including social sciences, natural sciences, and engineering (the majors and grades of 49 enrolled students are shown in Supplementary Table S1), completed the course, and all passed and earned credits. After the class, 48 students (97.96% of all enrolled students) participated in the university-organized end-of-term evaluation; the score of the course was 96.04, ranking 89/190 of all courses held by the Medical School, ranking 2,237/3,741 of all courses across the entire university. Twenty-two out of the 48 students provided subjective evaluations, mainly “good,” “learned more about public health,” “deepened my understanding of public health,” and “acquired some very practical public health knowledge,” etc. These evaluations suggest that students gained knowledge of public health from the classroom.

For the flipped classroom, 12 students provided 6 topics on public health as presentations, including “Debate on the Viewpoint of ‘Adequate Alcohol is Good for Health’,” “Water Security Issues,” “Viewing the Fukushima accident in Japan from a Public Health Perspective,” “Plague and Society in Jiangnan Region during the Qing Dynasty,” “Discussion on Hot Issues of Food Safety in Daily Life,” and “Advance on Cervical Cancer.” The rest of the students proposed 37 questions on public health and related topics (Supplementary Table S2). These questions are distributed in diverse aspects of public health, including the traditional and the frontiers, such as ‘the application of data science and artificial intelligence in public health,’ ‘globalization and public health equity,’ ‘rapid detection of food contaminants,’ etc.

The end-term paper from students also focused on a variety of subjects, such as “ancient and contemporary policies on infectious disease,” “insights and knowledge learned from the course,” and “food safety and food hygiene,” etc. Of note, 12 students suggested that the practical training of public health be enhanced, such as visiting local health departments, food and drug administration, community center, and campus canteen for field research.

A self-reported online questionnaire was deployed to gather feedback on the course, and students were free to choose whether or not to participate. The Institutional Review Board of Nankai University exempted the survey from ethical clearance as the survey is an anonymous survey conducted in an educational setting, approval number: NKUIRB2024100. Thirty-four undergraduates voluntarily participated in this anonymous survey, including 23 females and 11 males; 27 first-year students, 6 sophomores, and 1 junior. The participants represented various academic disciplines, including social sciences, natural sciences, and engineering.

### 4.1 Feedback on the current course

The evaluation parameters of the current course were analyzed first. For the survey on the rank of contents that the most and the least gained, 30 students provided validated data. The five parts of the contents are as follows: (1) Health and Medicine Knowledge; (2) Public Health Knowledge; (3) Public Health Methodology and Philosophy; (4) Proper View of Health Issues; (5) Values Education, which were required to rank from 5 to 1 for most and least gained. For the survey on the rank of difficulty and interests in the 4 segments, 33 students provided validated data. Students were required to rank from 4 to 1 for most and least difficult/interesting segments. Then, the number of rank were analyzed for calculating the “ranking score,” with a mean and standard deviation of the numbers of ranks within all the validated answers. The ranking scores were expressed with mean and standard deviation and were shown in Table 2. Kruskal–Wallis H test—a non-parametric statistical test for multiple groups—was employed to compare the ranking score between different contents.

**Table 2:** Students’ evaluation of the course in its current form.

Perceived satisfaction with the course content (*N* = 34)			
Satisfied		30 (88.24%)	
Basically satisfied		4 (11.76%)	
Neutral		0	
Mostly unsatisfied		0	
Completely unsatisfied		0	
Perceived difficulty of the course (*N* = 34)			
Very easy		4 (11.76%)	
Basically easy		14 (41.18%)	
Neutral		16 (47.05%)	
Basically difficult		0	
Very difficult		0	
Perceived interest in the course (*N* = 34)			
Interesting		24 (70.59%)	
Neutral		10 (29.41%)	
Boring		0	
Ranking score of modules based on perceived utility of the course (*N* = 30)			
	Mean	Standard deviation	Statistics
Health and Medicine Knowledge	4.16	0.91	Kruskal–Wallis H test between different contents *χ*^2^ = 66.62 *p* < 0.001
Public Health Knowledge	3.56	1.01	
Public Health Methodology and Philosophy	3.03	1.01	
Proper View on Health Issues	2.90	1.45	
Values Education	1.33	0.88	
Ranking score of segments based on perceived difficulty of the course (*N* = 33)			
Public Health Fundamentals and Population Research Methods	2.03	1.31	Kruskal–Wallis H test between different segments *χ*^2^ = 21.63 *p* < 0.001
Chemical Safety and Health	2.64	0.86	
Diet, Nutrition, and Health	2.15	1.12	
Immunology, Microbiology, and Infectious Diseases	3.18	1.01	
Ranking score of segments based on perceived interest of the course (*N* = 33)			
Public Health Fundamentals and Population Research Methods	2.58	1.20	Kruskal–Wallis H test between different segments *χ*^2^ = 9.16 *p* = 0.027
Chemical Safety and Health	2.54	1.00	
Diet, Nutrition, and Health	2.85	0.97	
Immunology, Microbiology, and Infectious Diseases	2.03	1.18	

The result showed that all surveyed students stated that the overall content of the course was “satisfied” or “basically satisfied.” Eighteen students out of the total 34 students (52.94%) stated that the overall difficulty of the course was “very easy” (*n* = 4, 11.76%) or “basically easy” (*n* = 14, 41.18%). There were 24 (70.59%) students who thought the course was “interesting.” For the five contents of teaching, students reflected that health/medical knowledge was the most gained content, with a score of 4.16 ± 0.91 (mean ± standard deviation), while values education was the least gained content with a score of 1.33 ± 0.88. For the interests and difficulty of the four segments, Immunology, Microbiology, and Infectious Diseases had the least interest (2.03 ± 1.18) and highest difficulty (3.18 ± 1.01).

### 4.2 Expectancy and suggestions

Students’ expectancy and suggestions on the course were also surveyed, and the result is shown in Table 3. Briefly, half of the students (*n* = 17, 50%) elected the course for health and medical knowledge, as the primary reason. Also, 29 (85.29%) students expected health and medical knowledge most in the course, and the students’ expectancy between these contents is statistically significant, as tested by the chi-squared test for frequencies between different contents. In addition, 18 (52.94%) students suggested that personal health and medical knowledge should be increased, higher than public health knowledge (*n* = 13, 38.24%), and public health methodology and philosophy (*n* = 8, 23.53%). For flipped classrooms, most students (32, 94.12%) suggested maintaining the status quo. Three students mentioned in the end-term paper that the optional PBL question or group presentation was a “friendly way” for students from different majors to participate in the flipped classroom.

**Table 3:** Students’ expectancy and suggestions on the content of the course.

The primary reason for electing the course (*N* = 34)		
For the Credit	7 (20.58%)	
For Health and Medical Knowledge	17 (50.00%)	
For Public Health Knowledge/Concepts	9 (26.47%)	
Other	1 (2.94%)	
Do you expect the following content in the course? (*N* = 34)		
Health and Medical Knowledge	29 (85.29%)	Chi-squared test between different contents *χ*^2^ = 19.91 *p* < 0.001
Public Health Knowledge	21 (61.76%)	
Public Health Methods	11 (32.35%)	
Public Health Concept	19 (55.88%)	
Biological Knowledge	20 (58.82%)	
Do you think the content should be increased/decreased? (*N* = 34)		
Personal Health/Medical Knowledge	Increase	18 (52.94%)
	Maintain	16 (47.06%)
	Decrease	0
Public Health Knowledge	Increase	13 (38.24%)
	Maintain	20 (58.82%)
	Decrease	1 (2.94%)
Public Health Methodology and Philosophy	Increase	8 (23.53%)
	Maintain	26 (76.47%)
	Decrease	0
Proper View on Health Issues	Increase	14 (41.18%)
	Maintain	20 (58.82%)
	Decrease	0
Values Education	Increase	5 (14.71%)
	Maintain	25 (73.53%)
	Decrease	4 (11.76%)
Do you suggest increasing/reducing the proportion of flipped classrooms? (*N* = 34)		
To increase	1 (2.94%)	
To maintain the status quo	32 (94.12%)	
To reduce	1 (2.94%)	

### 4.3 Attitude toward public health

Finally, students’ attitudes toward public health were also surveyed. As shown in Table 4, after taking the course, 27 (79.41%) students thought their majors were indirectly relevant to public health, and 2 students thought their majors were directly relevant to public health, significantly higher than their attitude before taking the course, as tested by the chi-squared test of independence. Most students (*n* = 23, 67.64%) stated that they were willing to devote themselves to public health in the future, although 16 of them stated being unable. Most of the students (*n* = 26, 76.47%) did not take other public health or health-related courses.

**Table 4:** Students’ association and attitude on public health.

Do you think your major is relevant to public health? (*N* = 34)			
	Before the course	After the course	
Irrelevant	13 (38.24%)	5 (14.71%)	Chi-squared test before and after the course *χ*^2^ = 6.306 *p* = 0.043
Indirect	21 (61.76%)	27 (79.41%)	
Direct	0	2 (5.88%)	
Are you willing to devote yourself to public health in the future? (*N* = 34)			
Yes		7 (20.59%)	
No		2 (5.88%)	
Unclear		9 (26.47%)	
Yes, but unable to do		16 (47.06%)	
Have you ever taken/are currently taking/plan to take other public health or health-related courses			
Yes		7 (20.59%)	
No		26 (76.47%)	
Unclear		1 (2.94%)	

## 5 Discussion and conclusion

Undergraduate public health education is regular in China, as a major named preventive medicine (25). Ongoing teaching and research related to public health are continuously being conducted based on the urgency of public health (26). However, few have focused on a public health course for non-medical students as a general elective course. In this study, we described for the first time a design and practice of public health as a general elective course for non-medical students, with knowledge, methodology, and philosophy on public health. The flipped classroom with PBL questions was also designed. Finally, the students’ feedback was collected and analyzed, showing particular demand for the teaching of public health from non-medical undergraduates. The results of this study hold significant importance for future public health curriculums and teachings, especially as a general education course for non-medical students.

One of the differences of the course, compared with other public health courses around the world, is that it was designed for non-medical students, and integrated more basic medical knowledge, but with less emphasis on certain areas of public health, such as community health, global health, and the health policy and management (13, 17, 27, 28), among others. Plus, as a general education elective, this course is modified to be more closely related to daily life and individual medical/health knowledge. By incorporating additional knowledge and content related to preventive medicine and other general medical knowledge, this course tends to be more specific and more accessible to undergraduate students, than other public courses. All lecturers involved in teaching this course have well-trained backgrounds in medicine. Interestingly, the survey showed that, basic biological or medical knowledge was considered as the most important content than other public health knowledge for non-medical students. As a general elective course, most students reflected that they did not take, or plan to take, any other public health or health-related courses (Table 4), meaning this course might be the only health-related course for the non-medical undergraduates, and the demand for biological or medical knowledge should be considered. Diet, nutrition, and health were the most interesting segments (Table 2), and should be considered increasing in the future. Previous studies usually focused on teaching public health to medical students (29, 30), and those courses may not particularly teach medical knowledge. However, as a general elective course for non-medical undergraduates, students are from different majors and are not required to take prerequisite courses, and therefore basic biological or medical knowledge should be incorporated. This is the difference between curricula for medical students and what teachers should pay attention to.

For the flipped classroom, the course provided two ways for students to participate, ①submit a PBL question related to public health to the teacher, along with the rationale for asking or a discussion about it; or ② work in groups of 1–5 people to present and discuss key issues on public health in class. In the survey, most students suggested maintaining the status quo of the flipped classroom (Table 4), and three students mentioned that the flipped classroom was “friendly” with a PBL question to the teacher as an alternative suggesting that the student-teacher cooperation PBL may be suitable for health-related course to non-medical students. PBL is an experiential learning approach that is widely adopted in diverse fields and educational contexts to promote critical thinking and problem-solving abilities (31). In PBL, students work in collaborative groups and learn what they need to know in order to solve a problem. The teacher usually acts as a facilitator to guide student learning (32). One of the advantages of PBL is that the students’ questions can drive motivation for knowledge (33). In the course, students were asked to propose a PBL question on the topic of public health, along with the rationale for asking. This may help not only students construct knowledge, but also teachers master the students ‘knowledge proficiency on public health.

One of the limitations of the curriculum is that it lacks internship and practice. We are endeavoring to establish cooperation with local centers for disease control and prevention, community centers, and the campus canteen for visiting and learning the future.

In conclusion, our study described a general education elective course in public health for non-medical undergraduates. The students’ feedback unveiled particular interests and gains from public health courses, providing evidence for public health teaching reforms in the future, especially public health teaching for non-medical students, and public health teaching in general education elective courses.

## Data Availability

The original contributions presented in the study are included in the article/Supplementary material, further inquiries can be directed to the corresponding author.
